# Decrease of Fecundity in Europe and America

**Published:** 1867-08

**Authors:** 


### ARTICLE VI.

### *Decrease of Fecundity in Europe and America.*

In a late number of the American Journal of Science and Art appears a paper which was originally read by Professor Storer of Boston before the American Academy of Arts and Sciences, about the close of the year 1858. Circumstances prevented its publication, but it is now brought before the people, who would do well to contemplate the startling facts it discloses. We shall go but slightly into details, confining ourselves to the general results.

It may be briefly stated that in Europe and America, there has been a marked and progressive decrease in the number of living births. In Sweden it has diminished by one ninth in sixty-one years, in Denmark by a quarter in 82 years; in Prussia by a third in 132 years, in England by two sevenths in a century; in Russia, by an eighth in 28 years; in Spain, by a sixth in 30 years; and in France by a third in 71 years. In France, for example, the rate of increase from 1801 to 1806 was 1.3 per cent. annually, while from 1806 to 1846 it had fallen to about a half of one per cent. The progressive character of this diminution may be seen by the following official figures, given in round numbers. The increase from

| | | |
|---|---|---|
| 1841—1846 | . . . . | was 1.200,000 |
| 1846—1851 | . . . . | " 380,000 |
| 1851—1856 | . . . . | " 256,000 |

In this country, it is quite as bad. Thus, in Massachusetts, the entire increase is due to the foreign population, the native population being either stationary or decreasing. In 1850, the population of that state was 994,665, and the births 27,664; in 1859 the population was 1,132.369, and the births 32,845. Thus every 36 people in Massachusetts in 1850 produced one birth, and in 1855, 34 produced one birth. The proportion of births in France, in 1850, was one to 37.

This occurs too in the face of the fact that marriages are increasing, and the comforts of life are becoming more generally diffused through all classes. It is time to use plain language on this subject. Dr. Storer's statistics explain the disgraceful fact. The diminution is voluntary. The curtain of this infamous rascality is lifted for us by a glance at his tables.

We learn there that in New York in 1805, there was one still-born child out of every 1633 people; while in 1849, there was one out of every 341 people living in that great city.

If we calculate the births in New York proportionally instead of by population, we reach the appalling result that in 1854 to 1857, one out of every eight infants was born dead. Now, as it is notorious that the public is acquainted with a very few of the cases of abortion, we are startled at the extent to which this murder of the innocents has been carried. Old Judea shuddered at one Herod. Modern America keeps one at every street corner. The horrible fact stares us in the face that child-murder has become a lucrative profession, and the very mothers themselves, are accessories before the fact in the villainous assassination.

A research into the proportions between abortions and still-births lifts still further the veil from this hideous mass of secret crimes. Tables of eminent obstetricians show that those proportions, in ordinary practice, are as 1 abortion to 78.5 still-births at full time. In New York, where it is certain that but few of the abortions were known, these proportions were as 1 to 4. The predominance in crime might well be supposed to be awarded to a great, rich, luxurious and dissolute city. But the State of Massachusetts, including the rustic population, has a far darker record. In that commonwealth, the proportion of abortions to still-births was 3 to 1 during the 14 years preceding 1855, while during the latter five years of that time, it was as 2 to 1. It is therefore 8 times worse than New York at its worst.

It must be borne in mind that we are not reading the records of illegitimacy; of women striving to hide their shame, at whatever cost to their offspring. These tables include the statistics of married life, and tell us that men and women, in comfortable circumstances, are in the habit of murdering their unborn children for base mercnaery reasons, to save the expense of a large and increasing household. It is a matter of simple justice to the Church of Rome to say that her communion furnishes but few and meagre items to this grand sum-total of rascality. She has set her face sternly and inflexibly against the monstrous villany, and the confessional enables her to enforce her righteous regulations in this respect. Hence the marked increase in population among her members.

---

